# Economic evaluation of wastewater surveillance in Ontario, Canada, using COVID-19 as a case study

**DOI:** 10.14745/ccdr.v52i06a04

**Published:** 2026-06-30

**Authors:** Yeva Sahakyan, David Champredon, Prakathesh Rabeenthira, Alex Rodriguez Barizonte, Manon Fleury, Beate Sander

**Affiliations:** 1 Health Systems and Policy Research Collaborative Centre, University Health Network, Toronto, ON; 2 National Microbiology Laboratory, Public Health Agency of Canada, Guelph, ON; 3 VHA Home HealthCare, Toronto, ON; 4 Faculty of Arts & Science, University of Toronto, Toronto, ON; 5 Public Health Ontario, Toronto, ON; 6 Institute of Health Policy, Management, and Evaluation, University of Toronto, Toronto, ON; 7 ICES, Toronto, ON

**Keywords:** economic evaluation, cost-utility, epidemic model, SARS-CoV-2, COVID-19, outbreak, wastewater surveillance

## Abstract

**Background:**

The COVID-19 pandemic has stimulated the use of wastewater surveillance (WWS) in Canada.

**Objective:**

To inform continued investment, the study assessed the cost-utility of WWS, alongside conventional surveillance, compared to conventional surveillance alone, using COVID-19 in Ontario as an example.

**Methods:**

This model-based cost-utility analysis measured WWS effectiveness by increased lead time of 1–10 days for public health response using the Ontario health system perspective. The model integrated SARS-CoV-2 transmission dynamics, SARS-CoV-2 RNA concentration in the sewage system, and disease progression. The analysis considered year-round surveillance with an outbreak occurring once in a decade, assuming WWS benefits accrue only in the outbreak year. At the individual-level, a lifetime time horizon was used and future health outcomes (quality-adjusted life years [QALYs]) and cost were discounted at 1.5%. The model was informed by population-based administrative data and was calibrated to real-world Ontario surveillance data.

**Results:**

For Omicron/BA.1-like outbreaks, a WWS program with a $15 million CAD/year budget maintained over 10 years would be cost-effective at a $50,000 CAD/QALY threshold if it detects an outbreak three or more days earlier, and cost-saving if it detects an outbreak 10 or more days earlier than conventional surveillance. For a less severe outbreak with lower transmission rates, e.g., XBB-like, the WWS program would be cost-effective if at least six outbreaks occur in a decade.

**Conclusion:**

The study findings suggest that long-term investments in WWS are likely cost-effective for low-frequency but high-impact outbreaks. Maintaining WWS infrastructure will enhance Canada's emergency preparedness for emerging and re-emerging pathogens.

## Introduction

As of December 2024, the COVID-19 pandemic has imposed substantial global burden with over 777 million cases, over seven million reported deaths (1) and a profound impact on health system expenditures (2,3). In Canada, as of September 2023, at least 374,106 hospital admissions have occurred due to COVID-19, of which 64,623 included intensive care unit (ICU) admissions and 46,472 resulted in death (4–7). The total expenditure incurred by the Canadian healthcare system was estimated to be over $9 billion CAD (4–7).

To guide public health interventions to mitigate the impact of COVID-19, surveillance data must be collected quickly and affordably (8). While polymerase chain reaction (PCR) testing is essential for individual-level detection, it has limitations for large-scale surveillance during major public health events (9). These include 1) reduced testing among individuals with mild or asymptomatic condition leading to underestimation of disease prevalence (10,11), 2) limited availability of PCR or point-of-care testing and capacity for laboratory personnel, as well as 3) the time lag between infection, testing and reporting that delays detection of community transmission (11).

The need for reliable and efficient population-level monitoring has highlighted the potential of wastewater surveillance (WWS) for tracking infectious disease prevalence and spread (12–14). Studies have shown that SARS-CoV-2 fecal viral load rises shortly after infection and peaks around symptom onset (15,16); therefore, PCR testing on wastewater samples may provide insights into infection trends in a population (17). Wastewater surveillance may complement conventional surveillance, by enabling earlier detection of community transmission, and capturing data from individuals with mild, presymptomatic and asymptomatic infection, often missed by conventional surveillance.

Beginning in September 2020, the Public Health Agency of Canada (PHAC) launched the National Wastewater Monitoring of Pathogens program, a systematic effort to collect and analyze wastewater from communities across Canada (18). As of early 2025, this program routinely samples from more than 90 municipal sites, covering an estimated 36.6% of the Canadian population. In Ontario, eight sites provide data for an estimated 31.1% of the provincial population. In addition to the federal program, many provinces and territories in Canada have also launched their own wastewater programs.

Wastewater surveillance implementation and maintenance requires resources; for example, the Ontario provincial WWS program reached a total operating expense of $15 million CAD in 2023–2024, which reflected a system with 59 monitored sites with sampling conducted three to five times per week (19,20).

To inform continued investment into WWS, this study assessed the cost-utility of WWS in addition to conventional surveillance compared to conventional surveillance alone, using COVID-19 in Ontario as an example. While the focus was on SARS-CoV-2, the framework may be applicable to other infectious pathogens that can be monitored through wastewater and is intended to inform long-term public health surveillance strategies.

## Methods

A model-based cost-utility analysis was conducted from the Ontario health system perspective, following Canadian guidance on economic evaluation in health (21,22). Because the timing of a future outbreak is unknown, the analysis considered year-round surveillance over 10 years with an outbreak occurring once in a decade. An outbreak was characterized by one major wave within the outbreak year. Individuals infected during the outbreak year were followed over their lifetime time to capture downstream health outcomes (i.e., life years, quality adjusted life years [QALYs] and cost, discounted by 1.5% as recommended (22)). Although WWS is maintained for each of the 10 years, the model conservatively assumed benefits from WWS occur only in the outbreak year, with no benefits during non-outbreak years.

### Population

The modelled population reflects epidemiological and demographic characteristics of the Ontario population who experienced COVID-19 infection, hospitalization, ICU admission, or death during selected waves of the recent COVID-19 pandemic or remained uninfected with COVID-19.

### Model structure and assumptions

**Figure 1:**
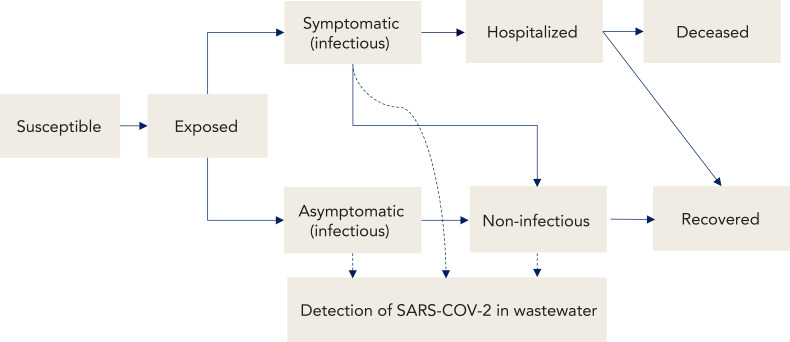
Model structure

The analysis was based on the epidemic model previously developed by the PHAC (23), which integrates both the transmission dynamics of SARS-CoV-2 at the population-level and the concentration of SARS-CoV-2 RNA in the sewage system following viral shedding by infected individuals. Disease progression was captured through several health states: susceptible; exposed (infected but not yet infectious); asymptomatically or symptomatically infected; hospitalized; recovered and no longer infectious but still shedding virus; fully recovered and permanently immune and no longer shedding the virus; and deceased (**Figure 1**). Infection occurred at a time-dependent transmission rate, which was simultaneously calibrated on clinical reports, hospital admissions and wastewater concentrations using real-world surveillance data from Ontario during the Omicron and XBB waves of the pandemic. The weekly clinical report and hospitalizations were retrieved from Public Health Ontario (24,25), and the SARS-CoV-2 RNA concentration in wastewater was sourced from the PHAC’s National Wastewater Monitoring of Pathogens program covering Toronto, Ontario. The key model parameters are summarized in **Table 1**.

**Table 1:** Key parameters

Transmission	Value	Source
Reproductive number^a^	Calibrated	Calibrated to match reported cases. (Gov. of ON) (24)
Average latency duration (not infectious yet), days	6	(PHAC, 2021) (26)
Symptomatic individuals who will not be hospitalized in future, days	12	(PHO, 2021) (27)
Average duration among symptomatic individuals who will be hospitalized in future, days	15	(PHO, 2021) (27)
Average duration among asymptomatic individuals, days	10	(PHO, 2021) (27)
Average duration of shedding after infectiousness, days	21	(Li, 2022), (Zhang, 2021) (28,29)
Proportion asymptomatic cases	0.33	(Oran, 2021), (Sah, 2021) (30,31)
Proportion of hospitalization^a^	Calibrated	Calibrated to match reported cases. (Gov. of ON) (25)
Average LOS in hospital, days	13	(CIHI, 2023) (5)
Days required to build immunity	40	(Carazo, 2023) (32)
**Time horizon**		
Individual level, years	Lifetime	(CADTH, 2017) (22)
Program level, years	10	Assumption
Case counting during the first wave of Omicron, days	120	Wave duration, (Gov. of Can, 2024) (18)
Case counting during the first wave of XBB, days	270	Wave duration, (Gov. of Can, 2024) (18)
**Age and life expectancy**		
**Individuals with COVID-19**		
Non-hospitalized	40	Statista Research Department (33)
Hospitalized (no-ICU)	65	(CIHI, 2023) (5)
ICU admitted	62	(CIHI, 2023) (5)
Died	84	(CIHI, 2023) (5)
Life expectancy	Age-dependent	Lifetables^b^
**Utilities**		
General population	Age-dependent	(Yan, 2024) (34)^b^
**Individuals with COVID-19, estimated 360 day average**		
Non-hospitalized	0.838	(Mao, 2024) (35)
Hospitalized (no-ICU)	0.827	(Mao, 2024) (35)
ICU admitted	0.746	(Mao, 2024) (35)
**COVID-19 attributable costs (2023 CAD)^c^**		
No hospital stay	$295	(Sander, 2025) (36)
Hospital stay (no-ICU), alive at end of follow up	$30,030	(Sander, 2025) (36)
Hospital stay (no-ICU), died before end of follow up	$30,610	(Sander, 2025) (36)
ICU stay, alive at end of follow up	$114,945	(Sander, 2025) (36)
ICU stay, died before end of follow up	$91,091	(Sander, 2025) (36)
Post-COVID-19 condition	$275	(Sander, 2025) (36)
Average annual costs for general population (2023 CAD)	Age-dependent	Estimated based on CIHI expenditures and lifetables^b^
WWS annual cost (2023 CAD)	$15 M	Gov. of ON (19)

Abbreviations: CAD, Canadian dollar; CADTH, Canadian Agency for Drug and Technology in Health; CIHI, Canadian Institute for Health Information; Gov. of Can, Government of Canada; Gov. of ON, Government of Ontario; ICU, intensive care unit; LOS, length of stay; PHAC, Public Health Agency of Canada; PHO, Public Health Ontario; WWS, wastewater surveillance

^a^ Reproductive number and proportion of hospitalization (of those who were symptomatic) were calibrated separately for the Omicron and XBB waves

^b^ Data available upon request from corresponding author

^c^ COVID-19 attributable healthcare costs inpatient hospitalizations, outpatient hospital visits, emergency department visits, publicly funded drugs (for everyone aged 65 years and select younger individuals based on means) physician services, rehabilitation services, complex care, homecare, long-term care, and other (e.g., laboratory tests/services, assistive devices), rounded to the nearest dollar, from index to 360 days of follow-up or death, whichever occurred first

### Strategies

The analysis assumed that WWS operated in addition to the conventional surveillance program and did not account for potential cost-savings from reduced reliance on patient-level testing. The impact of WWS relative to conventional surveillance was determined by increased lead time of one, three, five or 10 days for public health response (37,38) assuming that transmission would be reduced by 75% due to interventions/restrictions. This assumption was based on calibration of the epidemic model to the Omicron wave of the COVID-19 pandemic (November 2021–2022) in Ontario (24,25).

### Outcomes

Outcomes included the number of individuals who are asymptomatic, symptomatic and treated in the outpatient setting, hospitalized, admitted to ICU and died due to COVID-19. The analysis also considered life years, QALYs and health system cost associated with COVID-19 management. The incremental cost-effectiveness ratio, a measure of cost-effectiveness, was estimated assuming the annual cost of WWS to be $15 million CAD (19), along with the maximum expenditure at which a WWS program would still be cost-effective. Cost-effectiveness was assessed at the commonly used threshold of $50,000 CAD per QALY gained (39).

### Data

**Life expectancy:** Disease severity is reflected by the need for hospitalization and ICU admission. The median age of individuals who were hospitalized, including ICU admissions, was obtained from the Canadian Institute for Health Information (5). Individuals who required hospitalization were older, consistent with an age-related increase in disease severity. However, the analysis did not specifically address how hospitalizations and ICU admissions affected life expectancy beyond age-specific mortality. For individuals who were uninfected, asymptomatic and symptomatic but not hospitalized, the median age of the general Ontario population (33) was considered. Age-specific life expectancy was obtained from Statistics Canada (40) (Table 1; additional data available from corresponding author).

**Quality of life:** Health utility reflects the quality of life associated with a specific health state and is typically anchored at zero (death) and one (perfect health). Quality-adjusted life years are calculated as the product of utility and time spent in a specific health state (41). For uninfected and asymptomatic individuals, the utility values representative of the Canadian general population were applied, based on a study using the EQ-5D-5L instrument (34). For symptomatic individuals the mean utility 0–12 months post-infection was derived from a scoping review (35) and assumed the utility of the Canadian general population after 12 months (Table 1). The review included over 60 studies, mostly from Europe (N=39), Asia (N=15) and North America (N=8), with EQ-5D as the most frequently used instrument.

**Cost:** The annual cost-associated with WWS maintenance in Ontario was considered to be $15 million CAD (19). The COVID-19-attributable one-year costs (Table 1) were based on an Ontario population-based matched cohort study using health administrative data and included all publicly funded health services (36).

Zero COVID-19-attributable costs were assumed one year after infection with mean healthcare costs of the general population applied thereafter. Similarly, individuals with asymptomatic infections were assumed to incur healthcare costs equal to that of the general population. Lifetime healthcare costs for general population, were estimated considering life-expectancy and annual healthcare expenditures by age groups, based on Canadian Institute for Health Information data (40,42).

### Analysis

The base-case scenario considered deterministic values for costs and utilities, assuming the Omicron wave pandemic, with no post-COVID conditions. A year-round surveillance over a 10-year period was modelled, with an outbreak occurring either in the first year or in the last year. Benefits from WWS were assumed only occurred during the outbreak year. Additionally, to address parameter uncertainty, a probabilistic analysis was conducted, using beta distributions to sample utility values, gamma distributions to sample costs, and uniform distribution to sample the outbreak year.

A range of scenario analyses were conducted. One scenario assumed that 15% of the infected population would experience the post-COVID-19 condition, with reduced health utility of 0.005 and increased healthcare utilization continuing one year post infection for their remaining lifetime. For these individuals, a lifetime annual cost increment of $275 was applied, based on Sander *et al.* (36) who estimated the average net cost of $7.60 per 10-day interval during months 9–12 post-infection. Best- and worst-case scenarios were conducted using the upper and lower bounds of the confidence intervals from the transmission model predictions. The cost-utility of a WWS program maintained for 20 years, where an outbreak occurs during the 20^th^ year, was assessed as the most conservative scenario. Finally, the cost-effectiveness of the WWS was estimated assuming an outbreak with a lower transmission rate similar to the XBB wave, that may occur once or more frequently per decade.

## Results

### Scenario considering the Omicron wave

**Figure 2:**
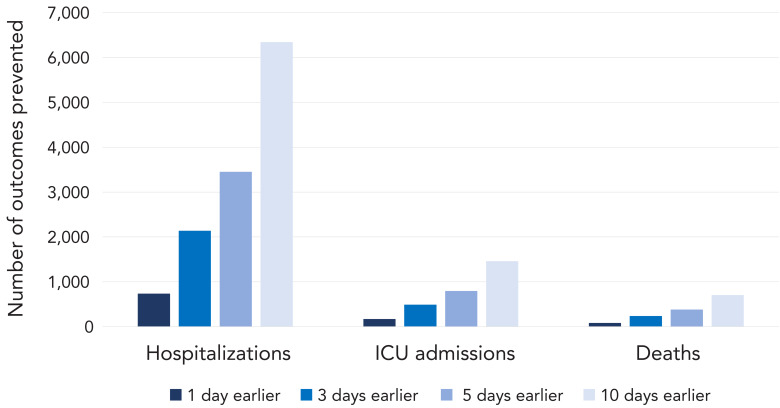
Hospitalizations, intensive care unit admissions and deaths averted per 14 million (i.e., Ontario) simulated population (base-case scenario)^a^ Abbreviations: ICU, intensive care unit; N, number ^a^ Comparator strategies provide 1, 3, 5 and to 10 days earlier detection of outbreaks, and, as such, 1 to 10 days earlier implementation of infection-control measures compared to conventional surveillance

Compared to conventional surveillance, a WWS program that detects Omicron/BA.1-like outbreaks one to 10 days earlier could prevent, on average, 735 (95% CI: 566–939) to 6,345 (95% CI: 4,794–8,071) hospitalizations, including 169 (95% CI: 130–216) to 1,459 (95% CI: 1,103–1,856) ICU admissions, and avert 81 (95% CI: 66–89) to 702 (95% CI: 557–795) deaths (**Figure 2**; additional data available from corresponding author), assuming a single wave of an outbreak once in a decade, and no other major events in the remaining nine years.

A WWS program with an annual budget of $15 million CAD (in addition to conventional surveillance) maintained over 10 years would be considered cost-effective at a $50,000/QALY threshold if it detects an outbreak three or more days earlier, and cost-saving if it detects an outbreak 10 or more days earlier than conventional surveillance, regardless of which year the outbreak occurs (**Table 2**). Probabilistic analysis showed that WWS providing a three-day lead time detection was cost-effective in 77% of simulations compared to conventional surveillance (data available from corresponding author).

**Table 2:** Expected quality-adjusted life years, and costs (2023 Canadian dollars) per person

Scenarios	QALY	Δ QALYs	Costs	Δ Costs	ICUR^a^	Max. annual program cost^b^
**Outbreak occurring during the 1^st^ year**						
Conventional surveillance	27.1267	Ref.	$296,863	Ref.	Ref.	Ref.
1 day earlier^c^	27.1269	0.00013	$296,861	−$2.20	$58,448	$13,308,549
3 days earlier^c^	27.1271	0.00039	$296,857	−$6.41	$9,250	$38,847,772
5 days earlier^c^	27.1274	0.00063	$296,853	−$10.36	Cost-saving	$62,949,902
10 days earlier^c^	27.1279	0.00118	$296,844	−$19.11	Cost-saving	$116,599,789
**Outbreak occurring during the 10^th^ year**						
Conventional surveillance	23.7248	Ref.	$259,635	Ref.	Ref.	Ref.
1 day earlier^c^	23.7250	0.00012	$259,633	−$1.93	$69,186	$11,639,553
3 days earlier^c^	23.7252	0.00034	$259,629	−$5.61	$12,926	$33,975,960
5 days earlier^c^	23.7254	0.00055	$259,625	−$9.07	1,737	$55,055,496
10 days earlier^c^	23.7259	0.00103	$259,618	−$16.71	Cost-saving	$101,977,270
**Outbreak occurring during the 1^st^ year, assuming lifetime post-COVID-19 condition**						
Conventional surveillance	27.1198	Ref.	$297,248	Ref.	Ref.	Ref.
1 day earlier^c^	27.1200	0.00019	$297,242	−$5.28	$24,982	$22,108,811
3 days earlier^c^	27.1203	0.00056	$297,232	−$15.42	Cost-saving	$64,588,401
5 days earlier^c^	27.1207	0.00090	$297,223	−$25.00	Cost-saving	$104,732,688
10 days earlier^c^	27.1214	0.00167	$297,201	−$46.32	Cost-saving	$194,308,753
**Outbreak occurring during the 10^th^ year, assuming lifetime post-COVID-19 condition**						
Conventional surveillance	23.7187	Ref.	$259,971	Ref.	Ref.	Ref.
1 day earlier^c^	23.7189	0.00017	$259,966	−$4.62	$32,553	$19,336,195
3 days earlier^c^	23.7192	0.00049	$259,957	−$13.49	Cost-saving	$56,488,515
5 days earlier^c^	23.7195	0.00079	$259,949	−$21.86	Cost-saving	$91,598,396
10 days earlier^c^	23.7202	0.00146	$259,930	−$40.51	Cost-saving	$169,940,927

Abbreviations: CAD, Canadian dollar; ICUR, incremental cost-effectiveness ratio; max., maximum; Ref., reference category; QALYs, quality-adjusted life years; Δ, incremental

^a^ Incremental cost-utility ratio has been calculated assuming the incremental cost for the wastewater surveillance program of $15 million annually

^b^ Wastewater surveillance program operational maximum annual cost for the strategy to remain cost-effective at a $50,000 CAD/QALY gained threshold, if maintained over 10 years

^c^ Wastewater surveillance program that provides 1 to 10 days earlier detection of outbreaks, and, as such, 1 to 10 days earlier implementation of infection-control measures compared to conventional surveillance

**Figure 3:**
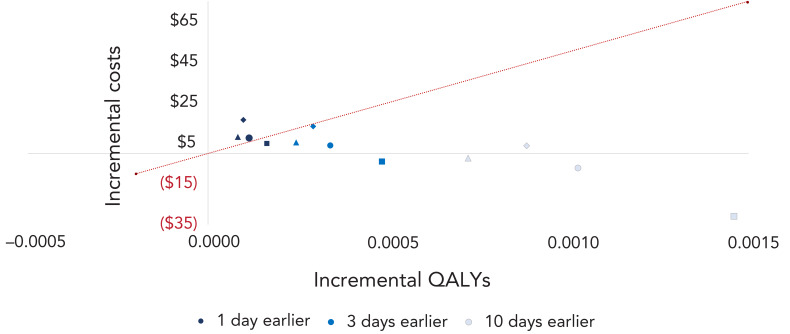
Incremental quality-adjusted life years and healthcare costs for scenarios involving Omicron-like outbreak^a,b^ Abbreviations: QALYs, quality-adjusted life years; WWS, wastewater surveillance ^a^ The graph illustrates the incremental costs in 2023 Canadian dollars (Y-axis) and incremental QALYs (X-axis) for WWS offering one, three and 10 days earlier detection of outbreaks, and, as such, one, three and 10 days earlier implementation of infection-control measures compared to conventional surveillance ^b^ Symbols in the figure represent different scenarios: ♦ (rhombus) denotes the WWS 20-year scenario, ● (circle) represents the base case scenario, ▲ (triangle) indicates the worst-case scenario and ■ (quadrant) accounts for the post-COVID condition. All scenarios assume a 10-year WWS maintenance period, with an outbreak occurring in the 10^th^ year, except for the “WWS for 20 years” scenario, the most conservative, where the WWS program is maintained for 20 years, and an Omicron-like outbreak occurs in the 20^th^ year. A strategy that detects outbreak five-day earlier and a best-case scenario were not plotted for better visualisation. The diagonal red line represents the $50,000 cost-effectiveness threshold, below which the WWS strategy is considered cost-effective

When lifetime disutility and costs associated with the post-COVID-19 condition are considered, a program that detects outbreaks three or more days earlier becomes cost-saving (**Figure 3**, Table 2). Summary for the outcomes for best- and worst-case scenarios are available from corresponding author. With a longer program horizon of 20 years, one outbreak in year 20^th^, and an annual budget of $15 million CAD, a WWS program would be considered cost-effective at a $50,000/QALY threshold if it detects an outbreak three or more days earlier than conventional surveillance (Figure 3, data available from corresponding author).

### Scenario considering the XBB wave

Compared to conventional surveillance, a WWS program that detects outbreaks similar to the XBB wave one to 10 days earlier could prevent 87 (95% CI: 63–98) to 901 (95% CI: 678–1,017) hospitalizations and avert eight (95% CI: 8–10) to 79 (95% CI: 79–101) deaths (data available upon request). For outbreaks like XBB, which have a lower transmission rate, WWS was not found to be cost-effective if the outbreak occurs only once per decade (data available upon request). A WWS program with an annual budget of $15 million CAD (in addition to conventional surveillance) maintained over 10 years and providing a three-day detection lead time, would be considered cost-effective if an XBB-like outbreak occurs in at least six out of the 10 years (data available upon request).

## Discussion

The study findings suggest that long-term investment in WWS is likely cost-effective for low-frequency but high-impact outbreaks. In scenarios with Omicron-like outbreaks, a WWS program with an annual budget of $15 million CAD maintained over 10 or even 20 years along with the implementation of public health policies that reduce transmission by 75% during the outbreak would be considered cost-effective at a $50,000/QALY threshold if it detects outbreaks three or more days earlier than conventional surveillance. For less severe outbreaks with lower transmission rates, like the XBB sub-variant, the WWS program was generally not cost-effective, unless outbreaks were frequent. However, these findings rely on the assumption of highly effective public health policies, which may not be feasible to sustain over multiple outbreaks. The observed differences between the two epidemiological scenarios are attributable to the substantially lower transmission rate of XBB, meaning that interventions, which limit transmission, would have a smaller impact compared to outbreaks with higher transmission rates.

The study findings align with studies showing that population-wide testing may not be cost-effective for pathogens with lower transmission rates. For example, Neilan *et al*., in their modelling study, showed that compared to testing of symptomatic cases only, PCR testing of the entire population was cost-effective when the virus’ reproductive number exceeded 1.6 (43). While this study focuses on WWS for population-wide monitoring, it similarly highlights the impact of transmission rates on cost-effectiveness. Unlike large-scale testing, which can be challenging to implement, WWS provides a more feasible approach. In Canada, for example, testing in March 2020 was limited to individuals with severe infection requiring hospitalization, long-term care residents and those with respiratory symptoms (44,45). Testing of selected population was later shown to be not cost-effective than testing all symptomatic individuals (43).

Cost-effectiveness data on WWS remains limited. Yoo *et al*. evaluated the cost-benefit of COVID-19 screening with antigen tests vs. WWS in a residential facility in Japan (46). They reported that WWS was economically justifiable at moderate, but not at high or low incidence levels; however, their model was constrained to a closed environment of 100 individuals and a program duration of just four days. In contrast, the current analysis considers the operation of WWS in Ontario, a province with a population of 14 M, over a 10- to 20-year time horizon, providing estimates of cost-effectiveness for a population-based surveillance program. Similarly, Mvundura *et al*., using an agent-based model, showed that WWS could avert 300–600 disability-adjusted life years over a six-month outbreak period and be cost-effective particularly in settings with high disease severity (47).

The effectiveness of WWS depends on several factors, including population density, wastewater infrastructure, pathogen detectability in wastewater, sampling frequency and analytical methods. Wastewater surveillance can complement conventional surveillance and enable community-wide monitoring regardless of healthcare access, healthcare-seeking behavior, testing disparities or socio-economic factors, providing a more affordable alternative to individual testing. Sanjak *et al.* compared the costs of WWS and clinical swab testing in 82 United States military bases under various outbreak scenarios, finding that WWS had $10.5 million–$18.5 million lower annual direct costs (48). Moreover, they estimated that over two-thirds of clinical swab testing could be replaced by WWS at no additional cost when accounting for lost work time due to swab testing requirements (48).

### Limitations

This study has several limitations. First, it focused solely on SARS-CoV-2, and a single outbreak within a ten-year period. This results in a conservative estimate, as Canada has experienced multiple waves of COVID-19 outbreaks spanning beyond one year (49). Second, the analysis did not incorporate the costs of downstream response measures that might follow WWS-triggered alerts; however, such public health responses would likely be implemented under conventional surveillance as well, albeit later, and therefore, incorporating these costs would likely have minimal impact on the overall cost-effectiveness conclusions. Third, the analysis did not account for the broader applicability of WWS in concurrently monitoring multiple pathogens, such as respiratory syncytial virus, influenza or enteroviruses (12,14,50). Detecting multiple pathogens using the same infrastructure could enhance public health preparedness at minimal additional cost. Furthermore, the study did not evaluate WWS from a broader societal perspective. Incorporating productivity losses would likely increase the value of WWS. Conversely, WWS may have limited value in detecting novel viruses during earlier waves of an outbreak, as assays development takes time, which can delay reliable detection.

### Strengths

This study has several strengths. This is one of the few economic evaluations of WWS based on a large scale, population-wide setting. The model was calibrated using real-world data on infections, hospitalizations and deaths, which strengthens the validity of the findings. By considering high- and low-transmission outbreaks and a broad range of scenarios, the study provides valuable insights for decision-making.

## Conclusion

Wastewater surveillance provides benefits that extend beyond the pandemic response, yet many of these benefits are difficult to quantify. This analysis focused on one measurable benefit for COVID-19 pandemic surveillance, where the most immediate and direct impact has been demonstrated. Wastewater surveillance appears to be cost-effective under various COVID-19 outbreak scenarios, especially for high-transmission events like Omicron/BA.1, with substantial health and economic benefits arising from early detection. However, this narrow scope likely underestimates WWS’s full potential. Beyond pandemic settings, WWS could enable year-round, population-wide surveillance of multiple pathogens, potentially reducing surveillance lag and providing critical lead time for public health response. Leveraging the existing WWS infrastructure will enhance Canada's preparedness for emerging and re-emerging pathogens.
